# Effects of different fertilizers on nutrient quality and mineral elements in different economic forage groups in Qilian Mountain alpine meadows

**DOI:** 10.7717/peerj.14223

**Published:** 2022-10-20

**Authors:** Xin Chen, Ting Jiao, Zhongnan Nie, Degang Zhang, Juan Wang, Juan Qi

**Affiliations:** 1 College of Grassland Science, Gansu Agricultural University, Key Laboratory of Grassland Ecosystem, Gansu Agricultural University, LanZhou, China; 2 Department of Economic Development, Jobs, Transport and Resources, Hamilton, Victoria, Australia

**Keywords:** Fertilization, Trace element, Qilian Mountains, Alpine meadow

## Abstract

The objective of this study was to examine the responses of nutrient quality and mineral elements of forages in different economic groups forages in the Qilian Mountains alpine meadows to different fertilization treatments. Eight fertilization treatments, potassium (K), nitrogen (N), zinc (Z), boric (B), copper (Cu), phosphorus (P), molybdenum (Mo), and selenium (Se), were sprayed onto forage roots, and no fertilizer was applied as a blank control (CK), with four replicates in each group. The forage trace element contents and nutritional quality of each economic group in to different treatments were determined in mid-August, and the trace element surplus and deficiency were analyzed. Compared with that in the control, the forage crude protein (CP) content in different economic groups under different fertilization treatments increased and fluctuated within the range of 7.7%–23.94%. The dry matter digestibility (DMD) ranged from 38.78% to 77.34% and showed no significant differences in DMD among treatments (*P* > 0.05). The DMD of legume forages was significantly higher than those of Gramineae grasses and sedges; there were no significant differences in forage acid detergent fiber (ADF) between any treatments and the control (*P* > 0.05), but ADF showed a decreasing trend in the treatments compared with that in the control. The P content increased by 81.81% in legumes (*P* < 0.05); however, it decreased by 38.46% and 61.29% in wilted grass and forbs (*P* < 0.05) and increased in Gramineae grasses and legumes under N treatment by 92.86% and 50% (*P* < 0.05), respectively. The Cu content under Cu, N, Zn, B and Se treatments increased significantly by 33.81%∼346.49% compared with that in the control. There were no significant differences in Zn content among the economic groups under different treatments (*P* > 0.05), but legumes showed better absorption of Zn. Consequently, the forages in this study were evaluated as having medium Cu (8.1–20 mg/kg), medium Se (0.11–1.0 mg/kg), and medium (30.1–60 mg/kg) to high (60.1–100 mg/kg) Zn contents. Comprehensive analysis showed that the application of different fertilizers could increase the CP and DMD of various forages, reduce ADF, and effectively improve the nutritional quality of pastures. The contents of N, Cu, Zn, and Se in forages increased under all treatments, and the contents of all forage elements were at appropriate levels. Applying K, N, P, Cu and Mo is beneficial to dominant species in Qilian Mountains alpine meadows.

## Introduction

Grasslands are the largest terrestrial ecosystem in China, accounting for approximately 12.4% of the global grassland area. They have high ecological value and important service functions (Yang et al., 2021; Kemp et al., 2013). Grassland health not only helps to maintain the stability of the grassland ecosystem but also improves its buffering capacity in response to ecological environmental deterioration and maintains ecological balance (Gao, 2012; Department of Animal Husbandry and Veterinary Medicine, 1996). The Qinghai-Tibet Plateau, known as the Earth’s Third Pole, has a total grassland area of 25 million hectares, accounting for 36% of the total grassland area in the country (Dong et al., 2017). Alpine grassland accounts for 50.9% of the total area of the Qinghai-Tibet Plateau, and this ecosystem hosts an important portion of global alpine grassland biodiversity (Liu, 2011). In recent years, the degradation of alpine grassland has gradually increased, while land productivity has gradually decreased, which also poses a serious threat to the normal provisioning of alpine grassland ecosystem services (Sun, Zhang & Dong, 2019). Consequently, grassland vegetation on the Qinghai-Tibetan Plateau has been degraded, grassland productivity has decreased, aboveground biomass has decreased, community structure and function have been destroyed, and community composition has become simplified (Sun et al., 2012; Zhu et al., 2021). Thus, the restoration and reconstruction of grasslands are important component of grassland ecosystem protection. In the case of environmental degradation, it is difficult to restore vegetation depending only on natural processes, and relief from human disturbance is necessary to restore grassland communities and balance grassland ecosystems.

Studies have shown that fertilization is an important method for enhancing grassland soil, accelerating herbage reproduction ability (Li, Wang & Zhao, 2004), improving grassland productivity, and restoring grassland community structure and composition (Yin et al., 2019). Zhang et al. (2019) showed that N addition could significantly increase community biomass and that the average plant height of gramineous plants was higher than that of plants in other functional groups. Wang et al. (2019) noted that important values associated with Gramineae grasses, sedges, and legumes in grassland plant communities increased significantly under P fertilization. The results showed that the reasonable application of fertilizer can not only promote growth and development and increase the number of tillers and grass yield but also enhance the nutritional quality of wild barley (Gong et al., 2019), *Leymus chinensis* (Liang et al., 2019), and other plants.

The nutrient composition of forage in natural alpine grasslands varies among different functional groups and families (Li et al., 2016). Grasses are a basic component of alpine grasslands and pastures. Research has shown that these species contain fewer macroelements and trace mineral elements than legumes and herbs (Wolański, Trąba & Rogut, 2015). To date, studies on the effect of fertilization on alpine grasslands have mostly concentrated on a large number of elements, and the understanding of this topic is therefore relatively well developed, but there have been few studies on the nutrient content of natural forage (Bai et al., 2016; Wang et al., 2017). Some authors have argued that the demand for catabolic nutrients (such as Zn and Cu) among herbaceous plants at a specific location is met by supply (Mills et al., 2013). In Yang et al. (2015) found that there were too many iron-containing compounds in the summer pasture of Yi Li, Xingjiang Autonomous Region. Zn and manganese were essentially sufficient, but Cu and Se were severely lacking, these patterns had no obvious relationship with altitude. Analysis of forage on northeastern Qinghai-Tibet Plateau showed that P, K, Zn and Cu were generally deficient in winter, while cobalt was insufficient in most areas, and all mineral element supplies were lower than the nutritional needs of livestock (Xin, 2010).

Studies have shown multiple trace element deficiencies in grazing livestock caused by deficiencies in certain trace elements in forages and soil in traditional grazing systems (Xin et al., 2011). To prevent pasture degradation, maintain pasture health and sustainability, and increase the demand for food and fiber from grazing systems by herdsman (Nie et al., 2013), it is imperative to develop appropriate pasture management guidelines for various regions of the Qinghai–Tibet Plateau. Fertilizing is an important measure (Xiong et al., 2019). There is little information on how macroelements and trace elements affect pasture growth, competition and plant nutrient levels, and there is a lack of data and research works on the status of trace elements in the Qilian Mountains. Therefore, in this study, the differences in element contents and nutritional qualities among different economic groups in the plant community (Gramineae grasses, legumes, sedges, wilted grass, and forbs) in degraded alpine meadows under treatments with N, P, K, Zn, Cu, B, Mo and Se fertilizer applications were addressed, and the natural forage of alpine meadows in the Qilian Mountains was selected as the research object. This study intends to provide the theoretical basis for artificial fertilization management in maintaining plant community diversity in natural grasslands and the rational management and utilization of pastures. In conclusion, this study sought to provide new insights into sustainable grassland ecosystem management in the alpine meadows.

## Materials & Methods

### Study site

The study site was located at the alpine meadow test station of Gansu Agricultural University in the Jinqiang River area of Tianzhu Tibetan Autonomous County, eastern Qinghai–Tibet Plateau (37°40′N, 102°32′E). The altitude was 2,040–4,874 m, the annual average temperature was −2 °C, and the monthly mean temperature was 12.4 °C in July (highest in the year) and −18.3 °C in January (lowest in the year). There was no absolute frost-free period, and the plant growth period reached 120–140 d. The long-term mean annual rainfall was 416 mm with most of the rain falling from July to September. The mean annual evaporation was 1,430 mm, 3.4 times the annual rainfall.

The pasture forage species in the region were diverse, and the major species included Gramineae grasses (predominantly *Elymus nutans*), legumes (*e.g.*, *Melissitus ruthenica*), sedges (*e.g.*, *Kobresia humilis*) and forbs (*e.g.*, *Potentilla bifurca, Gentiana straminea*). The soil was mountain black soil (chernozem) with pH = 7.5, the organic matter in the 0–40 cm soil layer reached 12%, and the contents of the measured elements were as follows: total nitrogen: 0.77%, total phosphorus: 0.11%, total potassium: 2.14%, hydrolysable nitrogen: 496 mg/g, and available potassium: 330 mg/g (Qi et al., 2015; Wang et al., 2017).

### Experimental design

The experiment was conducted at Tianzhu Grassland Station. Sample plots were established in flat and representative areas to enable fence protection, with an even mixture of the major species commonly distributed on the Qinghai–Tibet Plateau. A randomized complete block (RCB) design was used for eight nutrient treatments (K, N, Zn, B, Cu, P, Mo, and Se) and one blank control (CK), with four replicates of each treatment. Nine treatments were applied to the selected grazing plots, for 36 plots in total.

Every plot was 1 m × 1 m =1 m^2^ in size, leaving a 50 cm gap between plots and replicates. The experimental layout is given below (Table 1). The fertilizer applied in each quadrat was dissolved in a spray pot containing 100 mL of distilled water, and then sprayed onto the roots. Then, the pot was washed with an equal amount of distilled water for every treatment, and the rinse water was sprayed onto the plot. The CK plot was not fertilized, but the same amount of water was sprayed to reduce the effect of additional water on the growth of plants.

### Experimental materials

Fertilizer resources, types and application amounts are shown in Table 2. The application rate was determined based on recommendations for each nutrient and the soil conditions at the site. All fertilizers were supplied by Damao Chemical Company, Tianjin, China. Each fertilizer was weighed and applied evenly to individual plots based on their designed application rates and fertilization method.

**Table 1:** The experimental layout.

Rep 1	3-1 Zn	5-1 Cu	8-1 Mo	7-1 Se	9-1 CK	2-1 N	6-1 P	1-1 K	4-1 B
Rep 2	1-2 K	9-2 CK	6-2 P	2-2 N	7-2 Se	3-2 Zn	4-2 B	5-2 Cu	8-2 Mo
Rep 3	8-3 Mo	4-3 B	5-3 Cu	1-3 K	6-3 P	9-3 CK	3-3 Zn	7-3 Se	2-3 N
Rep 4	5-4 Cu	8-4 B	7-4 Se	9-4 CK	2-4 N	4-4 B	1-4 K	6-4 P	3-4 Zn

**Table 2:** Fertilizer types and dosages.

Treatment	Resources	Element content (%)	Fertilizer amount (kg/hm)	Fertilizer amount (g/m)
K	KCl	60.00	55.00	5.50
N	CO(NH_2_)_2_	46.00	282.60	28.26
Zn	ZnSO_4_⋅ 7H_2_O	22.73	5.00	0.50
B	Na_2_B_4_O_7_⋅ 10H_2_O	11.34	8.00	0.80
Cu	CuSO_4_⋅ 5 H_2_O	25.45	16.00	1.60
P	Na_2_HPO_4_	8.66	232.56	23.26
Se	Na_2_SeO_3_	45.66	0.35	0.035
Mo	Na_2_Mo_4_⋅ 2H_2_O	39.65	10.00	1.00

### Sampling design and measurements

The fertilization treatments were carried out in early May, and sampling was carried out in August. The upper part of the pasture was mowed 2 cm from the ground when each plot (1 m ×1 m) was sampled. All samples in each quadrat were classified according to their functional group (Gramineae grasses, legumes, sedges, wilted grass, and forbs), transported to the laboratory, placed in an oven for 15 min at 105 °C and then dried at 65–75 °C for 7–8 h to a constant weight. The samples were ground and then sieved with a 40-mesh sieve for further analysis.

For Zn and Cu content determination, samples were ashed in a muffle furnace at a high temperature of 550 °C ±15 °C for 6–8 h to obtain residue. Then, the residue was dissolved with hydrochloric acid solution, diluted to volume, and then directed into the air acetylene flame of an atomic absorption spectrophotometer. The details of the determination method can be referenced in Lu, 2000. For P and Se, samples were accurately weighed in crucible, after heating with a muffle furnace plus acid digestion, the contents of P and Se were determined by graphite furnace atomic absorption spectrometry (Yuan et al., 2016).

For crude protein concentration, N was determined by small-scale Kjeldahl digestion (Liu, 2015). The samples were digested with concentrated sulfuric acid under the catalyst until the solution became clear. The sample was then distilled with a Kjeldahl instrument. After distillation, the N content in the sample was determined by titration. The N concentration was multiplied by 6.25 to calculate the crude protein content (Korn, Santos & Ferreira, 2005; Qi et al., 2015).

For ADF, 0.5 g of the ground herbage from each sample was placed into a nylon bag (400 mesh) tied with a nylon thread. The nylon bag was then put into acidic detergent and boiled for 1 h over a low flame. The nylon bag was then washed with warm water, and soaked with a small amount of acetone. The nylon bag was oven dried at 80 °C until constant weight to determine ADF (Li et al., 2020).

For DMD, the samples were accurately weighed and placed in a homemade nylon bag, which was sealed with cotton rope and placed in a trachea. Then, the collected rumen fluid and artificial culture medium were mixed evenly at a volume ratio of 1:2. After incubation for 48 h, the nylon bags were oven dried to a constant weight, and DMD was calculated (Menke et al., 1979).

### Data analyses

Excel was used for data collection, and all responses were tested with analysis of variance (ANOVA) using Statistical Product and Service Solutions (SPSS 26.0). Differences among treatments were separated using Duncan’s multiple range test and differences were declared when *P* < 0.05.

The gray correlation analysis method (Wu, Xie & Sheng, 1990) was used to comprehensively analyze the relevant indicators of each treatment. According to the principle of correlation analysis, the value of the correlation is positively related to the performance of comprehensive traits.

## Results

### Effect of different fertilization treatments on CP in the evaluated plant groups in alpine meadows

**Figure 1:**
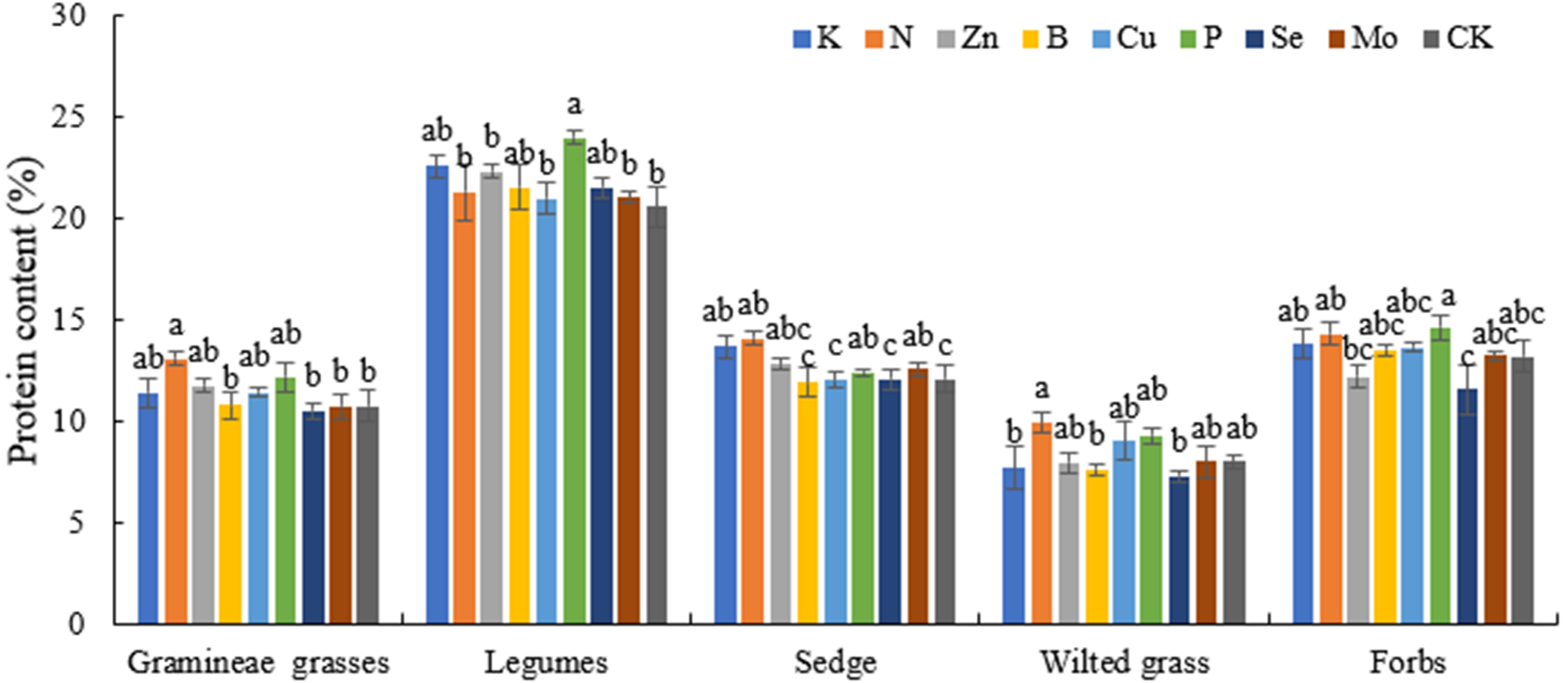
Effects of different fertilization treatments on plant CP in alpine meadow.

Gramineae grasses and sedges showed significant differences in the N treatment compared with the CK (*P* < 0.05), with maximum values of 13.06 and 14.09 and increased of 21.49% and 14.52%, respectively. The CP content in legumes under the N treatment increased by 12.39% compared with that under the P treatment (*P* < 0.05). In comparison to values obtained under CK, the CP content in legumes and sedges under P treatment increased by 16.55% and 2.66%, respectively (*P* <  0.05), indicating that P treatment promoted the CP content in legumes, The CP content in Gramineae grasses, legumes, wilted grass, and forbs under K, Zn, B, Se, and Mo treatments was 9.93%, 8.57%, 4.77%, 4.67%, and 2.24% higher than that in CK, respectively, indicating that K, Zn, B, Se, and Mo treatments can effectively increase the CP content. The application of N, P, K, and Mo, significantly improved the CP in sedges, there were no significant differences among treatments for forbs or wilted grass. The application of N significantly improved the CP contents in grass forages, indicating that grass forages are very sensitive to N (Fig. 1).

### Effects of different fertilization treatments on the forage ADF content in alpine meadows

There were no significant differences in ADF contents in different plant functional groups under K, N, Zn, B, Cu, P, Se, and Mo treatments compared with CK (*P* > 0.05), while in legumes, the lowest ADF content under Cu treatment was 29.95, 12.92% lower than that in CK (Table 3).

### Effects of different fertilization treatments on the DMD of forage in alpine meadows

The DMD of Gramineae grasses under K and N treatments was 14.22% (*P* > 0.05) and 13.76% (*P* > 0.05) higher than that in CK. Overall, the DMD of legumes was higher than that of Gramineae grasses and sedges, indicating that legumes have high nutritional value for livestock (Table 4).

### Effects of different fertilization treatments on the plant P content in alpine meadows

Compared with the under CK, the P content in legumes under the P treatment increased by 81.81%, while that in wilted grass and forbs decreased by 38.46% and 61.29%, respectively. The P content of Gramineae grasses and legumes under N treatment increased by 92.86% and 50%, respectively, compared with that in CK (Table 5).

**Table 3:** Different fertilization treatments on plant ADF content in alpine grassland (%).

Treatment	Gramineae grasses	Legumes	Sedges	Wilted grass	Forbs
K	40.78 ± 1.29a	30.79 ± 1.24a	37.74 ± 0.62a	44.32 ± 0.87a	39.42 ± 1.56a
N	41.54 ± 0.60a	31.76 ± 1.99a	39.24 ± 0.73a	42.26 ± 0.57a	39.23 ± 2.05a
Zn	40.93 ± 1.92a	31.42 ± 0.96a	37.93 ± 1.64a	44.82 ± 2.00a	42.98 ± 1.79a
B	38.48 ± 0.60a	32.33 ± 4.69a	37.67 ± 2.47a	44.69 ± 1.44a	40.81 ± 1.17a
Cu	40.30 ± 0.71a	29.95 ± 2.92a	38.59 ± 2.21a	42.48 ± 3.01a	39.84 ± 2.17a
P	42.40 ± 0.99a	32.67 ± 2.27a	40.20 ± 2.29a	43.55 ± 1.51a	36.14 ± 1.00a
Se	40.92 ± 2.17a	32.86 ± 4.41a	37.42 ± 1.83a	45.56 ± 1.55a	40.00 ± 3.05a
Mo	42.52 ± 2.00a	31.43 ± 1.51a	38.48 ± 1.01a	45.14 ± 1.79a	38.90 ± 1.33a
CK	42.21 ± 2.13a	33.82 ± 3.24a	37.59 ± 2.76a	43.33 ± 1.89a	42.88 ± 4.37a
*P*-value	0.697	0.994	0.980	0.883	0.581

**Notes.**

The data are the mean ± standard error, with significant differences in the same column indicated by lowercase letters (*P* < 0.05); the same as below.

**Table 4:** Different fertilization treatments on the forage DMD of plants in alpine grassland (%).

Treatment	Gramineae grasses	Legumes	Sedges	Forbs
K	47.56 ± 1.96a	74.07 ± 3.02a	51.97 ± 0.64a	61.74 ± 1.36a
N	47.37 ± 2.10a	70.20 ± 0.63a	47.34 ± 2.43a	61.57 ± 2.59a
Zn	41.45 ± 4.10a	69.33 ± 4.55a	46.21 ± 1.88a	55.85 ± 1.39a
B	40.39 ± 2.27a	68.35 ± 3.82a	48.45 ± 3.64a	52.64 ± 4.19a
Cu	38.78 ± 2.18a	71.17 ± 2.57a	53.45 ± 4.13a	58.69 ± 3.75a
P	40.16 ± 1.67a	71.21 ± 4.06a	49.42 ± 1.70a	63.26 ± 4.18a
Se	40.19 ± 3.35a	77.34 ± 2.35a	50.77 ± 2.58a	61.44 ± 3.16a
Mo	39.50 ± 2.90a	71.67 ± 0.49a	50.40 ± 2.88a	60.32 ± 4.55a
CK	41.64 ± 4.08a	75.72 ± 2.07a	44.80 ± 2.27a	59.09 ± 1.88a
*P*-value	0.289	0.463	0.474	0.414

**Notes.**

The data are the mean ± standard error, with significant differences in the same column indicated by lowercase letters (*P* < 0.05); the same as below.

**Table 5:** Different fertilization treatments on plant P content in alpine grassland (%).

Treatment	Gramineae grasses	Legumes	Sedges	Wilted grass	Forbs
K	0.18 ± 0.0205b	0.25 ± 0.0266cd	0.12 ± 0.0067de	0.30 ± 0.0405ab	0.72 ± 0.0787a
N	0.27 ± 0.0434a	0.33 ± 0.0070b	0.35 ± 0.0108a	0.35 ± 0.0159a	0.48 ± 0.0449b
Zn	0.09 ± 0.0115c	0.19 ± 0.0078ef	0.14 ± 0.0189de	0.28 ± 0.0093bc	0.26 ± 0.0065c
B	0.09 ± 0.0016c	0.25 ± 0.0082cd	0.08 ± 0.0014f	0.14 ± 0.0128ef	0.26 ± 0.0176c
Cu	0.08 ± 0.0039c	0.23 ± 0.0277de	0.10 ± 0.0056ef	0.21 ± 0.0116cde	0.33 ± 0.0297c
P	0.12 ± 0.0075c	0.40 ± 0.0089a	0.24 ± 0.0214c	0.26 ± 0.0143bcd	0.31 ± 0.0156c
Se	0.18 ± 0.0058b	0.28 ± 0.0024bc	0.29 ± 0.0228b	0.22 ± 0.0086cd	0.25 ± 0.032c
Mo	0.11 ± 0.0126c	0.17 ± 0.0116f	0.15 ± 0.0038de	0.19 ± 0.0223de	0.24 ± 0.0141c
CK	0.14 ± 0.003bc	0.22 ± 0.007de	0.21 ± 0.0115c	0.36 ± 0.0437a	0.5 ± 0.0286b
*P*-value	<0.001^∗∗^	<0.001^∗∗^	<0.001^∗∗^	<0.001^∗∗^	<0.001^∗∗^

**Notes.**

The data are the mean ± standard error, with significant differences in the same column indicated by lowercase letters (*P* < 0.05); the same as below.

### Effect of different fertilization treatments on the Cu content of plants in alpine meadows

**Figure 2:**
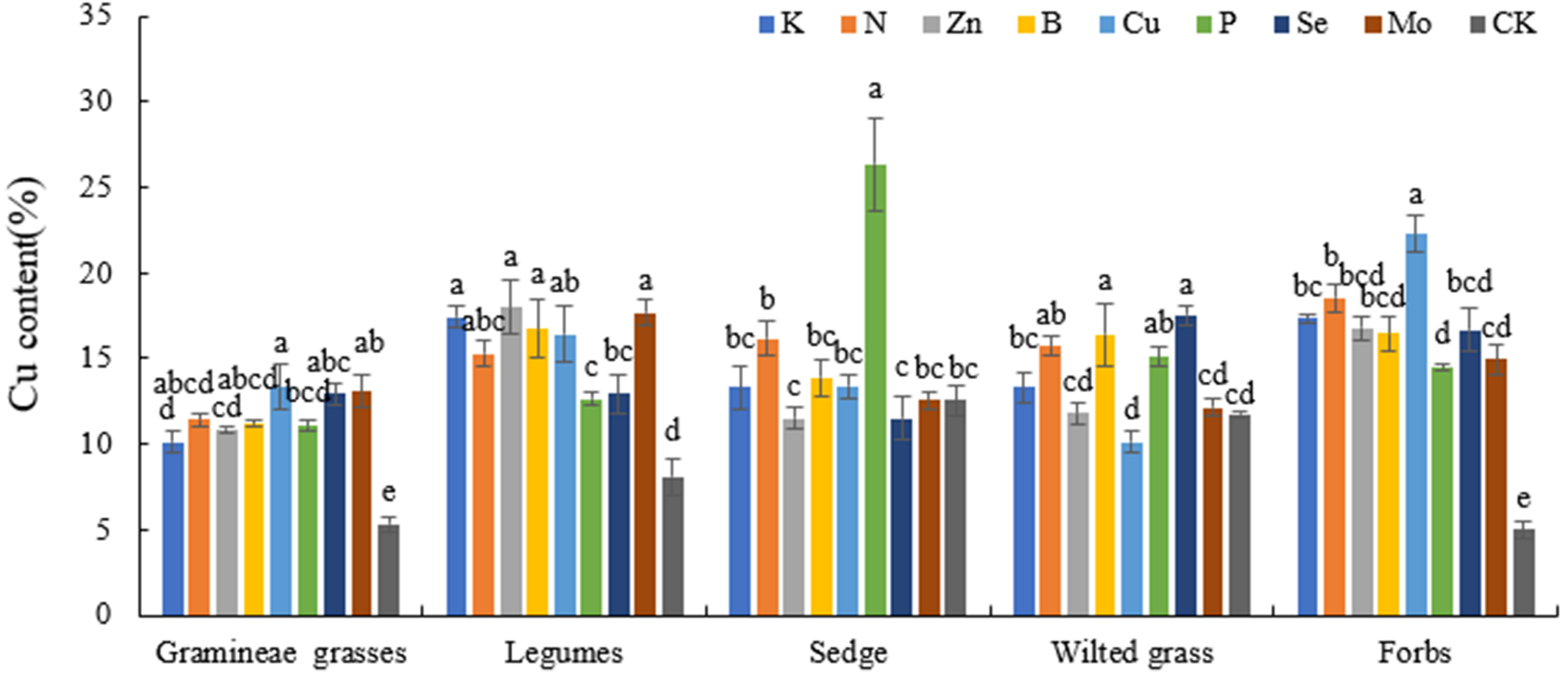
Effects of different fertilization treatments on plant Cu in alpine meadow.

The Cu content in the K, N, Zn, B, Cu, P, Se, and Mo treatments was significantly increased compared with that in CK. In addition, the Cu contents in Gramineae grasses, legumes, and forbs under Zn, B, Se, and Mo treatments significantly differed from those in CK (*P* < 0.05). The Cu content in Gramineous grasses increased by 103.56%, 110.32%, 142.96%, and 145.78% under Zn, B, Se, and Mo treatment, respectively, compared with CK, indicating that these elements can significantly increase the Cu content (Fig. 2).

### Effects of different fertilization treatments on the Se content in plants of forage alpine meadows

**Figure 3:**
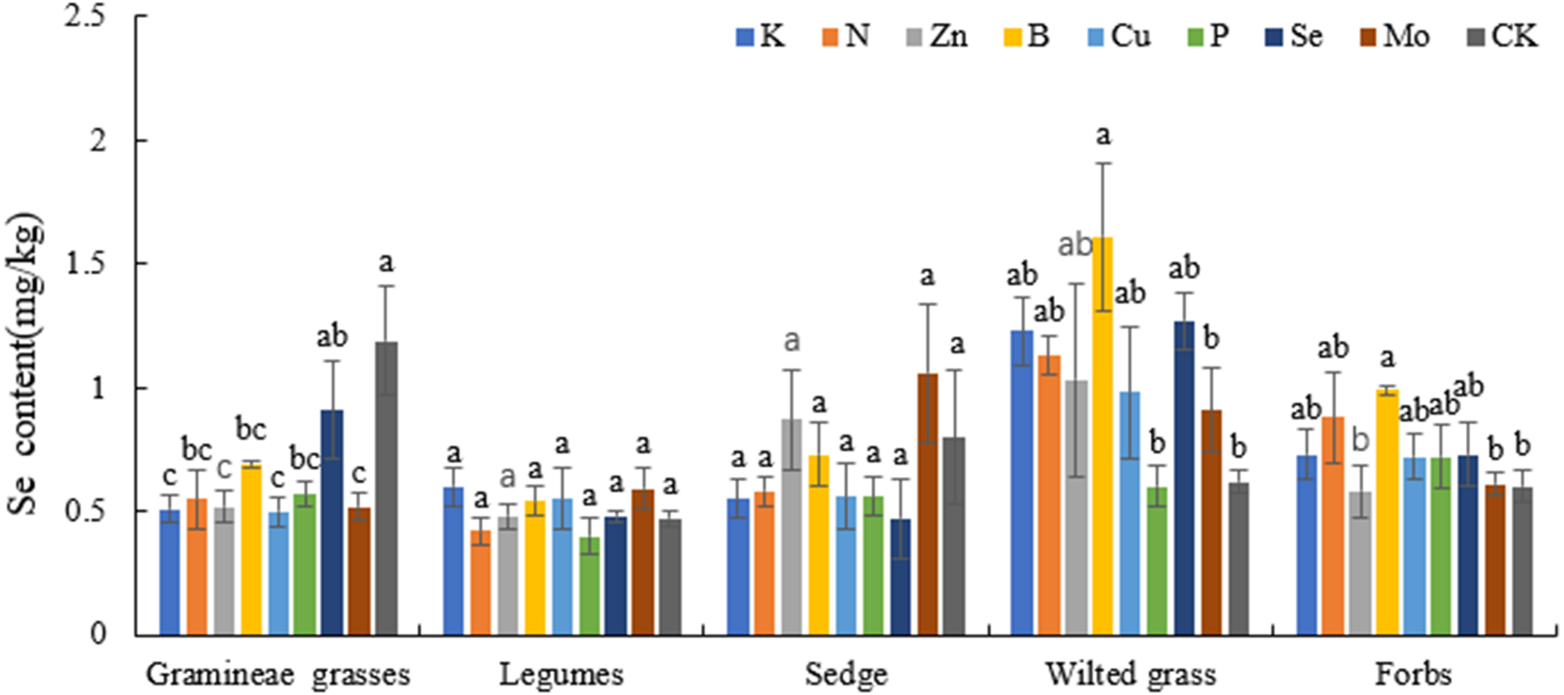
Effects of different fertilization treatments on plant Se in alpine meadow.

Compared with that in CK, the Se content in Gramineae grasses, wilted grass and forbs significantly increased by 97.35%, 102.21%, and 53.94% (*P* < 0.05), respectively, while that in legumes and sedges did not significantly differ from that in CK (*P* > 0.05). The Se content in each treatment (except the P treatment) significantly differed from that in the CK (*P* < 0.05) (Fig. 3).

### Effects of different fertilization treatments on the Zn content of forage plants in alpine meadows

**Figure 4:**
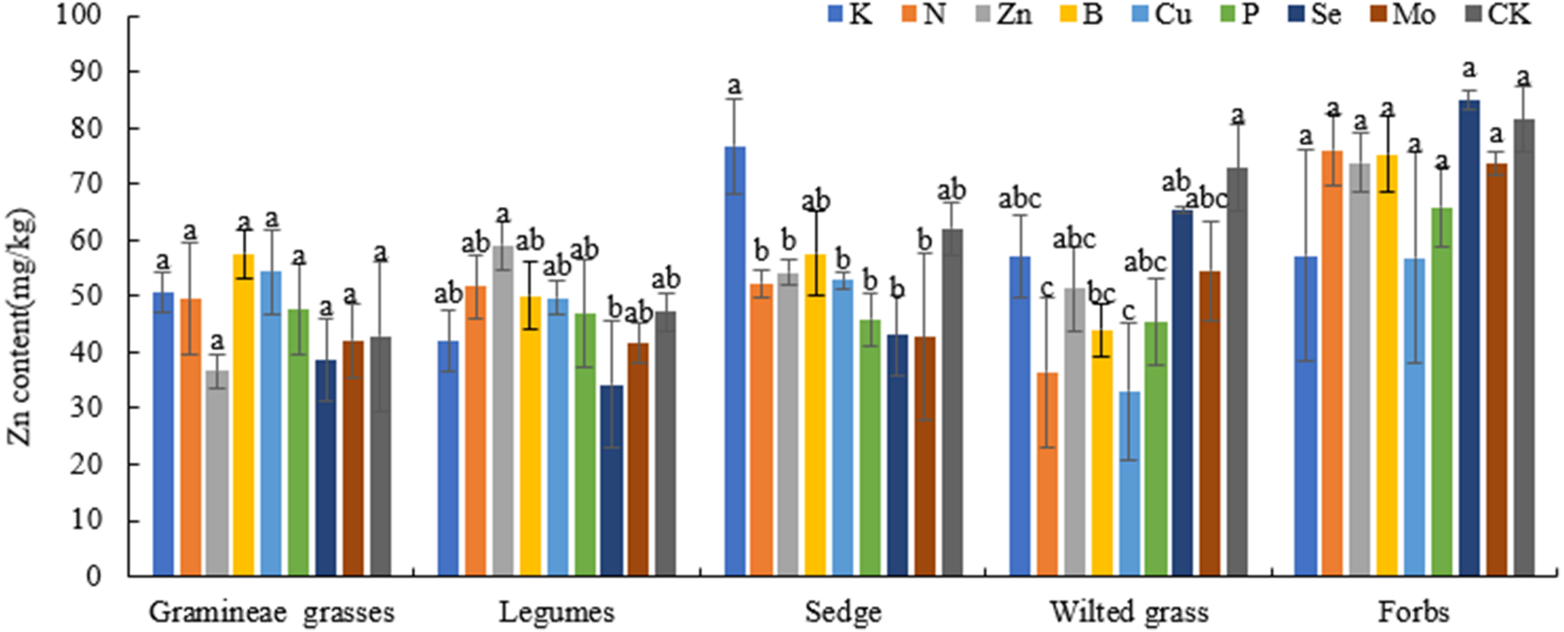
Effects of different fertilization treatments on plant Zn in alpine meadow.

Under different fertilization treatments (Fig. 4), there were no significant differences in the Zn content of Gramineae grasses, legumes, sedges, wilted grass, and forbs compared with that in CK (*P* > 0.05). However, under the Zn treatment, compared with CK, the Zn content in legumes increased by 25.01% (*P* > 0.05), while it decreased in other economic groups, which showed better absorption of Zn by legumes.

### Comprehensive analysis of gray correlations

To further clarify the effects of different fertilizers on the nutritional quality and mineral element content in different plant economic groups of alpine grasslands, a comprehensive gray correlation analysis was carried out on the dominant taxa: Gramineae grasses, legumes and sedges. The larger the correlation value is, the closer the relationship between the sample sequence and reference sequence, indicating that the treatment effect is stronger. The indexes for Gramineae grasses and sedges under the N fertilizer treatment were high, with the highest comprehensive ranking order. Legumes under the K treatment showed the highest ranking. The correlation values were in the order N > Cu >B > K > Se > CK > P > Mo > Z for Gramineae grasses, K > Zn > P > Cu > Mo > B > N > Se > CK for legumes and N > K > P > Mo > CK > Se > Zn > Cu > B for sedges. Overall, the effect of applying K, N, P, Cu and Mo was beneficial for dominant species in the Qilian Mountains alpine meadow (Table 6).

**Table 6:** Correlation degrees and ranking of indexes of dominant species under different fertilization treatments.

Dominant species	Treatments	ADF	DMD	CP	P	Zn	Cu	Se	Weighted relevance	Sort
	K	0.8590	1.0000	0.7280	0.5070	0.7420	0.5863	0.3740	0.6852	4
	N	0.8233	0.9888	1.0000	1.0000	0.7138	0.7046	0.3901	0.8029	1
	Zn	0.8516	0.7278	0.7700	0.3443	0.4861	0.6472	0.3787	0.6008	9
	B	1.0000	0.6953	0.6606	0.3358	1.0000	0.6819	0.4510	0.6892	3
Gramineae grasses	Cu	0.8841	0.6507	0.7280	0.3333	0.8621	1.0000	0.3731	0.6902	2
	P	0.7882	0.6883	0.8330	0.3883	0.6659	0.6688	0.3959	0.6326	7
	Se	0.8521	0.6894	0.6382	0.5126	0.5117	0.9204	0.5919	0.6738	5
	Mo	0.7834	0.6697	0.6556	0.3646	0.5625	0.9490	0.3785	0.6233	8
	CK	0.7955	0.7343	0.6600	0.4127	0.5761	0.3638	1.0000	0.6489	6
	K	0.9125	0.8707	0.8335	0.4315	0.4966	0.8976	1.0000	0.7775	1
	N	0.8334	0.7556	0.7211	0.6141	0.6957	0.6518	0.4898	0.6802	7
	Zn	0.8592	0.7338	0.8064	0.3581	1.0000	1.0000	0.5922	0.7642	2
	B	0.7948	0.7105	0.7382	0.4309	0.6518	0.8033	0.7373	0.6952	6
Legumes	Cu	1.0000	0.7816	0.6959	0.3982	0.6419	0.7670	0.7775	0.7232	4
	P	0.7742	0.7825	1.0000	1.0000	0.5795	0.4899	0.4619	0.7268	3
	Se	0.7631	1.0000	0.7368	0.4990	0.4047	0.5030	0.5874	0.6420	8
	Mo	0.8584	0.7956	0.6994	0.3333	0.4904	0.9456	0.9371	0.7228	5
	CK	0.7138	0.9313	0.6677	0.3853	0.5878	0.3417	0.5703	0.5997	9
	K	0.9783	0.9324	0.9285	0.3740	1.0000	0.4367	0.4417	0.7274	2
	N	0.8922	0.7700	1.0000	1.0000	0.5455	0.4980	0.4568	0.7375	1
	Zn	0.9662	0.7387	0.8081	0.3865	0.5655	0.4047	0.6786	0.6498	7
	B	0.9835	0.8037	0.7101	0.3333	0.6034	0.4469	0.5467	0.6325	9
Sedges	Cu	0.9269	1.0000	0.7221	0.3504	0.5501	0.4376	0.4455	0.6332	8
	P	0.8471	0.8356	0.7587	0.5449	0.4866	1.0000	0.4475	0.7029	3
	Se	1.0000	0.8840	0.7243	0.7052	0.4652	0.4049	0.4059	0.6557	6
	Mo	0.9330	0.8704	0.7795	0.4062	0.4634	0.4222	1.0000	0.6964	4
	CK	0.9888	0.7030	0.7258	0.4847	0.6657	0.4223	0.6091	0.6571	5

## Discussion

Although the content of trace elements in organisms is very small, they play a vital role in the structure and function of ecosystems (Lynch & Clair, 2004). The application of trace elements can promote plant growth, yield, and quality (Ma et al., 2019; Yu, Cao & Wu, 2013). P, Cu, Se, Zn, and other elements are essential nutrients for plants and livestock, and forage is a direct source of various nutrients for livestock. A lack or an excess of any elements may lead to unhealthy plants or prevent their growth and development, thus decreasing grassland biomass and forage nutritional quality, according to previous studies (Saurabh et al., 2017; Yuan et al., 2016; Zhang et al., 2021). CP and DMD are important indicators with which to evaluate feed value and nutritional quality, which have direct impacts on livestock productivity and economic value (Huo et al., 2006). However, ADF could affect forage digestion by livestock (He, Yang & Zhang, 2019).

The study showed that when nitrogen application rates are increased within a certain range, plant CP increases (Oikehs, Kling & Okoruwa, 1998). In this study, the CP contents in Gramineae grasses and sedges under N treatment were 21.49% and 14.52% higher, respectively, than in those without any fertilizer. Based on the different responses of CP to N fertilizers in different plant groups in the alpine meadow, we found that Gramineae grasses exhibited the strongest response, followed by sedges. Under nutrient sufficiency, the high nutrient utilization efficiency of Gramineae grasses and sedges, results in little competition and adaptation to nutrients under the conditions of nutrient limitation. According to the CP grade index of forage chemical composition, forages were classified as superior, medium, and inferior (≥16% as superior, 10%–15% as medium, ≤10% as inferior) (Ren, 1998). In this study, legumes were in the top forage category, while Gramineae grasses, sedges, and forbs were in the middle forage category, and the CP content was found to be significantly higher in legumes than in Gramineae grasses and other types of forage. He (2015) showed that the forage DMD in August was between 31.14% and 68.25%, and consistently, it was between 38.78% and 77.34%. in this study. The contents of CP and fiber in the forage determines its DMD (Shi et al., 2013). In this study, with the increase of in CP, he DMD of forage also increased, and the DMD of legumes was higher than that of Gramineae grasses and sedges. This explains the were a positive correlation between CP and DMD, and livestock can benefit from legume forage due to its high nutritional value. This result also comprehensively indicates that an appropriate amount of fertilization in the grassland can allow the rational use of the maintain forage healthy growth and development, enhance the forage nutritional quality, so allowing grassland rational using.

P can improve forage quality, and its content changes with the plant growth period. P also plays an important role in animals (Wang et al., 2019; Lu, 2012). The P content in Gramineae grasses, legumes, and sedges under N treatment was higher than that in CK, which indicated that N could promote P absorption. Cu plays an important role in photosynthesis, especially in the leaves of many plants. Reasonable application of Cu could induce resistance in plants, reducing the environmental stress effects caused by a lack of Cu (Ji, 2019). According to previous research, Cu content in most plants is 2–20 mg/kg, and it was also within this range in different plant economic groups evaluated in this study. The Cu content in Gramineae grasses, legumes, and forbs under Zn, B, Se, and Mo treatments was significantly different from that in the CK (*P* < 0.05), indicating that Zn, B, Se, and Mo treatments can significantly promote Cu absorption. A suitable level of Zn in forage is 20–60 mg/kg (Li & Wang, 1995a; Li & Wang, 1995b). In this experiment, the Zn content in Gramineae grasses and legumes was 36.6–59.03 mg/kg, and that in sedges, forbs, and wilted grass was higher than the appropriate level. The Zn content of forage in the Zn treatment was also at an appropriate level. Previous research showed that high levels of Zn in plants may inhibit metabolic functions and result in retarded growth of both roots and shoots (Fontes & Cox, 1998). This indicated that the forage Zn content in the Qilian Mountains alpine meadow could meet the Zn demand for normal plant growth and development and thus ensure that the Zn demand for cattle and sheep growth is met. Therefore, there is no need to supplement Zn in the Qilian Mountains area. Se is an indispensable trace element for plants that can increase plant resistance and antagonism (Wang, 2010); it plays a role in nutrient and ion balance, and it maintains a certain ratio with other nutrients or ions in the plant body, which is promotes plant metabolism, growth and development (Sharma, 1999). Appropriate amounts of Se can increase the chlorophyll content of forage leaves, enhance plant photosynthesis, and improve the soluble protein content in plant tissues, which explain why the application of Se promotes plant growth. It was illustrated that an appropriate amount of Se promoted the growth of plants (Chen & Liu, 1996). Research and analysis have shown that the Se content in forage grass is relatively low (Li et al., 2016), ranging from 0.1–1.0 mg/kg, and the area where the Se content in forage was 0.1 mg/kg was a normal area (Li & Wang, 1995a; Li & Wang, 1995b). In this study, the Se content in each plant economic group was 0.46–1.27 mg/kg, which was higher than the normal level, showing that there was no lack of Se in this area.

Differences among plant economic groups in an alpine meadow can also lead to heterogeneity in the mineral element content of forages, even in the same region, the contents of trace elements in different types forage are distinct. According to classification and evaluation indicators of trace elements in forages developed by Fu, Zhou & Zhang (2000), the forage Cu content in Qilian Mountain alpine meadows was evaluated as medium (8.1–20 mg/kg), the Se content as medium (0.11–1.0 mg/kg), and the Zn as medium (30.1–60 mg/kg) to high (60.1–100 mg/kg) level. Under all fertilization treatments, all element contents in forage increased and were at an appropriate level, which indicated that element fertilization could improve the forage quality and value, thereby improving the livestock production performance (Mills et al., 2013). According to the results of this experiment, different fertilization treatments can help maintain plant community diversity and rational grassland use.

Under the comprehensive influence of global climate change and human activities, forage growth and development were disturbed, which affected plant demand for trace elements, resulting in their surplus and deficiency in plants. The results of this study showed that gray system theory could be used to comprehensively evaluate the effects of various fertilization treatments on the nutritional quality and mineral element content of plants of different economic groups in alpine grasslands. Different plant economic groups had varying sensitivities to different types of fertilizer. The Qilian Mountain alpine pasture is most sensitive to the application of N, K, P, Cu, and Mo fertilizers, and these fertilizers had the best effects. This result also showed that applying fertilizers to such pastures could improve the nutritional value of the pastures and prevent their livestock from lacking mineral elements and becoming diseased, which could help maintain pasture and livestock health and support the rational utilization of grasslands.

However, many factors affect the trace element content of forage grasses, making it difficult to evaluate feed trace elements. The soil, forage, and livestock are interdependent in the material cycling of alpine meadow grazing systems. Soil is a key source of mineral elements required for forage grasses, which are a central supplier of mineral element requirements for livestock. Disturbance to grasslands, to varying degrees, induces imbalances in the nutrition of some essential minerals in these ecosystems. Therefore, it is necessary to conduct further investigations on the relationship between different plant economic taxa and the role of soil trace elements in high- meadow productivity.

## Conclusions

As a result of global climate change and human disturbance, and as plants grow and develop, their demand for trace elements is affected, resulting in changes in the trace element content of alpine meadow plants. This study showed that the effects of applying macroelements and microelements to alpine meadows varied among plant economic groups. The nutrient utilization efficiency was higher under nutrient sufficiency. There is little competition and adaptation to nutrients under nutrient limitation.

The scientific application of fertilizers can make plants resistant, which is beneficial to plant metabolism, growth, and development. In this study, we applied a fertilization treatment to a mountain region, and the effects of applying K, N, P, Cu, and Mo were good for the dominant species in the Qilian Mountain alpine grassland. The Zn and Se contents of forages in this area can meet livestock needs without supplementation. Therefore, appropriate fertilization of forage grasses can make them reasonably useable.

Fertilization can improve the management of pasture grazing, and the results can help develop more effective fertilization strategies. An appropriate amount of fertilization can maintain healthy forage growth and improve its nutritional quality. These findings can provide a theoretical basis for the rational utilization and effective microelement fertilization of Qilian Mountain alpine meadows. However, since the study was conducted in only one location, it is necessary to study more areas in the alpine meadow. In the future, such work will enable more convenient and efficient management and protection of alpine meadows.

##  Supplemental Information

10.7717/peerj.14223/supp-1 Supplemental Information 1 Raw data

## Additional Information and Declarations

### Data Availability

The following information was supplied regarding data availability: The raw measurements are available as a Supplementary File.
